# Predictive Capability of QSAR Models Based on the CompTox Zebrafish Embryo Assays: An Imbalanced Classification Problem

**DOI:** 10.3390/molecules26061617

**Published:** 2021-03-15

**Authors:** Mario Lovrić, Olga Malev, Göran Klobučar, Roman Kern, Jay J. Liu, Bono Lučić

**Affiliations:** 1 Know-Center, Inffeldgasse 13, 8010 Graz, Austria; mlovric@know-center.at (M.L.); rkern@know-center.at (R.K.); 2 Ruđer Bošković Institute, P.O. Box 180, 10002 Zagreb, Croatia; olga.malev@irb.hr; 3 Department of Biology, Faculty of Science, University of Zagreb, Rooseveltov Trg 6, 10000 Zagreb, Croatia; goran.klobucar@biol.pmf.hr; 4 Institute of Interactive Systems and Data Science, TU Graz, Inffeldgasse 16c, 8010 Graz, Austria; 5 Department of Chemical Engineering, Pukyong National University, Busan 608-739, Korea

**Keywords:** predictive QSAR, toxicity, ToxCast, zebrafish embryo, rdkit, structural descriptors, structural fingerprints, machine learning, imbalanced classification, aquatic toxicology

## Abstract

The CompTox Chemistry Dashboard (ToxCast) contains one of the largest public databases on Zebrafish (*Danio rerio*) developmental toxicity. The data consists of 19 toxicological endpoints on unique 1018 compounds measured in relatively low concentration ranges. The endpoints are related to developmental effects occurring in dechorionated zebrafish embryos for 120 hours post fertilization and monitored via gross malformations and mortality. We report the predictive capability of 209 quantitative structure–activity relationship (QSAR) models developed by machine learning methods using penalization techniques and diverse model quality metrics to cope with the imbalanced endpoints. All these QSAR models were generated to test how the imbalanced classification (toxic or non-toxic) endpoints could be predicted regardless which of three algorithms is used: logistic regression, multi-layer perceptron, or random forests. Additionally, QSAR toxicity models are developed starting from sets of classical molecular descriptors, structural fingerprints and their combinations. Only 8 out of 209 models passed the 0.20 Matthew’s correlation coefficient value defined a priori as a threshold for acceptable model quality on the test sets. The best models were obtained for endpoints mortality (MORT), ActivityScore and JAW (deformation). The low predictability of the QSAR model developed from the zebrafish embryotoxicity data in the database is mainly due to a higher sensitivity of 19 measurements of endpoints carried out on dechorionated embryos at low concentrations.

## 1. Introduction

The Computational Toxicology Chemistry Dashboard (CompTox) [1] provides data that have been modeled for determination of Mode of Action (MoA), hazard identification, compound screening, and prioritization, as well as risk assessment for roughly 8000 unique compounds [2]. To date, the CompTox effort has been successful in giving new perspectives to chemical characterization, toxicity testing, and exposure modeling. The CompTox database is a respectable source of toxicity data of chemicals created by the US Environmental Protection Agency (US EPA). It allows for a shift to simultaneous evaluation of numerous chemicals based on disruption of different biological target and signaling pathways using high-throughput screening data and computational approaches [3]. As a part of CompTox, a zebrafish (ZF) developmental toxicity assay has been used to evaluate potential ecotoxicological and harmful effects on humans’ health. These animals are easy to rear and maintain, they mature rapidly and are sufficiently small for sustaining testing in 96-well microtiter plates reducing the cost by means of small amounts of test chemicals [4]. Physiological, molecular, and functional features such as rapid development, optical transparency during the whole embryonic development, well characterized embryonic ontogenesis and ex utero development, availability of genomic data and ~70% of genetic similarity amongst humans and zebrafish make this model appropriate for evaluating a broad range of chemical-biological endpoints across vertebrate taxa [5,6]. Zebrafish assays have been subjected to numerous QSAR studies. Many of these focus on relatively small sets of molecules [7,8,9,10] and close-to-mechanistic models. QSARs developed on large data sets on zebrafish embryo toxicity are rare [11,12,13]. The ZF embryo assays consist of up to 1092 compounds (by ID) tested on diverse developmental malformations. Such data sets are valuable for creating models on broad chemical spaces and low concentrations, which are key for evaluating the risk due to many novel compounds present in mixture at nano-to-micro-concentration ranges in fish [14] and in river surface water and sediment [15], with often unknown MoA and synergistic actions.

Several reports present QSAR models on the subsets of the CompTox ZF library, namely the NHEERL_ZF_144hpf_TERATOSCORE assay, described originally in [4] that uses zebrafish embryos to screen 309 Phase 1 environmental compounds, which are mainly pesticides and antimicrobials. Models developed on the NHEERL_ZF assay are reported also in the literature [11,12]. However, both studies involved only one zebrafish toxicity endpoint (50% mortality data) and the models were developed and validated on data sets of less than 300 compounds from Padilla et al. [4]. They showed reasonable prediction quality for the self-defined toxicity cutoffs, having Matthew’s correlation coefficients on the test sets of 0.89 (*n* = 58) [11] and 0.77 (*n* = 61) [12]. Furthermore, these studies present a high importance of the LogP descriptor in the models which should not be neglected. Nevertheless, this appears expected given that assays where the chorion (membrane around the embryo) was kept will show association of toxicity with hydrophobicity [5]. It should be noted that the chorion can serve as a permeability barrier to larger molecules [16] and their penetration can vary due to physiochemical properties of compounds, their cationic charge or electrostatic attraction between chemicals and the chorion [17]. Another set of 19 “Tanguay_ZF” CompTox assays [18] were conducted on dechorionated embryos that are reported to be more sensitive than the chorionated ones [19], and represent a bigger challenge in models as reported here and in [20]. It has been demonstrated that with the use of chorion-intact embryos the sensitivity to identify teratogens was higher while the specificity was lower compared to data obtained with dechorionated embryos [21]. The use of dechorionated embryos may involve different agreements between sensitivity and specificity [17], due to the higher mortality of embryos. Dechorionation of embryos is desirable as it removes a potential barrier to chemicals, thus allowing a more effective evaluation of toxicity mechanisms that underlie effects of chemical exposure [22]. 

The concept of evaluating the predictability or modelability, essentially based on distance and similarity measures, has been developed in several studies [23,24,25,26]. The distance or similarity between molecules was estimated from the predictions of activities done by previously developed QSAR models. For every compound in a data set, and on a given set of structural features, the Euclidean distance to its first nearest neighbor was calculated. After that, it is estimated whether its first nearest neighbor compound belongs to the same or to a different activity class, and the total number of those belonging to each class was counted. Then, the modelability index for classification QSAR endpoint is defined as the ratio of compounds having the first nearest neighbor in the same class to the total number of compounds in the data set [24]. Later, also analogous modelability index based on the Euclidean distance measured between compounds in feature space and activity prediction by classification QSAR models was introduced for classification endpoints [25]. An alternative and conceptually simpler method for estimating modelability is the one used by Thomas et al. [26] which is based only on the consideration of predictive capabilities of models comparing with the gain of the model over the level of random (chance) accuracy. Namely, they considered two sets of features for 309 compounds and 84 classification algorithms to analyze modelability of 60 ToxCast phase I endpoints measured for approximately 300 compounds in the five-fold cross-validation procedure. The final result was that all endpoints are of low modelability. As the main evaluation metrics, the Area Under the Curve (AUC) of the Receiver Operating Characteristic (ROC) curve was used, which has the value of 0.5 in case of random models. The approach applied in this study for analysis of modelability of 19 CompTox endpoints related to zebrafish toxicity is analogous to the one introduced and used by Thomas et al. [26] with the differences in validation methodology and the model evaluation metrics for classification models. Namely, we evaluated the real predictive capabilities of QSAR models on external (never seen) test data set, which is a more objective measure of real model’s (endpoints’) predictivity or modelability. All mentioned methodologies [23,24,26,27] have applied three-fold or five-fold cross-validation which is a less strict validation procedure than the validation on external test set involving 20% of chemical compounds of the complete data set, as used in this study.

Our aim is to assess the use of the “Tanguay_ZF” CompTox embryo assays for QSARs studies and the building of predictive models for future utilization of chemical compounds and risk assessment (ERA). Majority of published literature on use of QSAR in ERA relies heavily on acute lethal effects (LC50) predictions while sublethal effects are equally or even more important for toxicity assessment of polluted aquatic environment and especially fish organisms. It is therefore of great importance to assess the usefulness of such data on toxic effects on fish organism for possible use in risk assessment of polluted aquatic ecosystems. Additionally, the “Tanguay_ZF” set is conducted on whole organisms, thus the complexity of reactions to specific chemicals is included unlike the majority of the ToxCast data which are based on cell-based in vitro assays. To achieve this, we have tested three different classifiers and chemical representations in a large experiment matrix of 209 model runs (11 models × 19 targets) to obtain results independent of machine learning, chemical representation (i.e., descriptors and fingerprints) and model hyperparameters. The prediction results are reported on train and test sets by means of multiple evaluation metrics such as the Matthew’s correlation coefficient (MCC) [28], Cohen’s Kappa [29] and Real-Accuracy (RA) (previously named $\Delta Q_{2}$ [30]), which were selected due to their ability to capture performance in imbalanced datasets [31,32,33]. Additionally, the same methodology was applied on a 6–7 times larger set of toxicities from the Tox21 US EPA database. In such a way, the validity of the applied methodology was confirmed through obtained higher values of predictive quality parameters which are completely comparable to the corresponding results of other authors.

## 2. Results

The accuracies and qualities of obtained QSAR models are reported by means of MCC, Real-Accuracy, Balanced Accuracy (BA), and Accuracy across the 209 models. Interested readers are referred to the Supplementary Materials (Table S1) for a full confusion matrix of each model. All model combinations are indexed based on the options they include, i.e., algorithm, scaling, predictive data set, target, and feature selection (see Section 4). To understand the relationships between diverse metrics, we have correlated the values of different quality parameters obtained on the Test set for all models. 

The values of the Pearson correlation coefficients are given in Table 1. The results show that MCC and Cohen’s Kappa correlate almost perfectly (0.97), while both Kappa and RA correlate above 0.84 with MCC. BA shows negative correlation with all the three afore mentioned metrics (<−0.19) so does Accuracy (<−0.18). 

**Figure 1:**
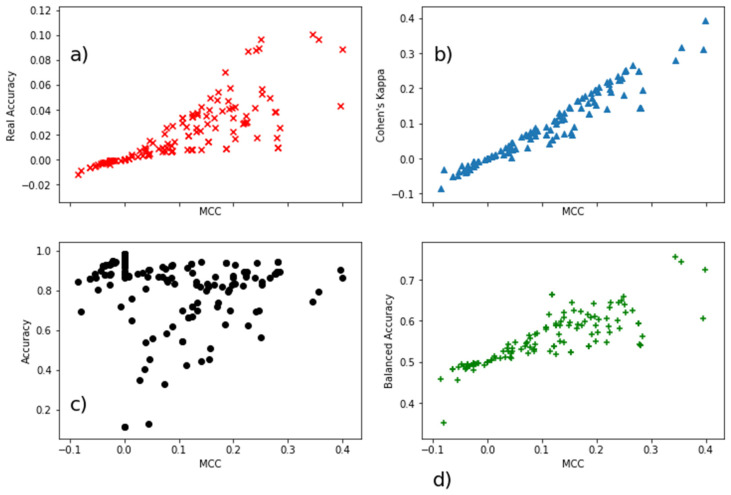
Scatter plots of values of four model quality parameters against MCC corresponding to 209 models on the respective test sets (**a**) Real Accuracy, (**b**) Cohen’s Kappa, (**c**) Accuracy and (**d**) Balanced Accuracy.

The comparison of different model quality metrics is also shown in Figure 1. Both BA and Accuracy have high values even for models with MCC values close to 0, which are considered here as random models. Therefore, reporting the model quality only with the Accuracy or BA appears inappropriate. Since MCC highly correlates (0.99) with the Cohen’s Kappa, we have transferred the categorization [29] which considers a Cohen’s Kappa score below 0.20 as to “slight agreement” (just above random, which is 0), to results reported by MCC and defined a threshold of below 0.20 MCC as slight correlation (just above random). 

Scatter plots of model quality parameters for all models (11 models for each of 19 endpoints) are presented in Figure 1. We aim to reveal the model’s predictability independently of the set of options considered in the model optimization process. The distributions show that quality metrics of most of models on the endpoints perform in the region defined previously as almost random models (the MCC axis in Figure 1 does not pass MCC 0.4). The criteria being set, 26 out of 209 have MCC Test values above 0.20, while 19 models surpass the same threshold with MCC during cross-validation in model training (MCC CV) (see Table S1). There are eight models which satisfy both criteria (MCC CV and MCC Test > 0.20). Overall, four out of these models are related to the endpoint ActivityScore, three on MORT and one on JAW, and these models have Real-Accuracy values (%) on the test set between 3 and 9%, i.e., all being above the level of the random accuracy. The correlation between Cohen’s Kappa and MCC is higher for positive values of both parameters (Figure 1). In addition, these two parameters are identical for models having FN = FP (see Table 3 for definitions).

**Figure 2:**
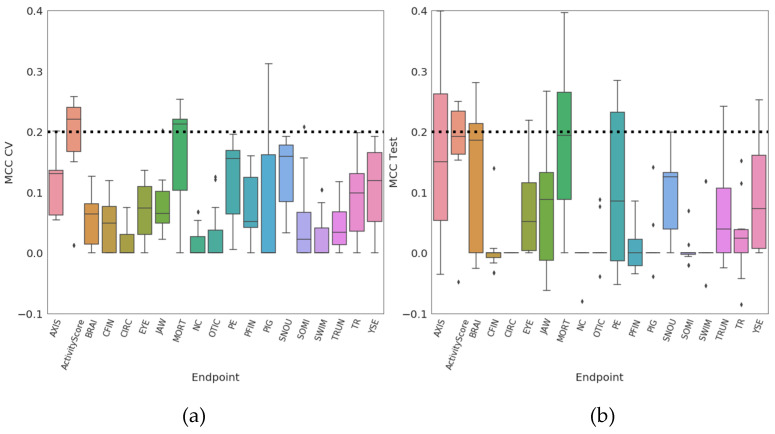
Boxplot diagrams of MCC CV values for the training set (**a**) and MCC Test values for the test set (**b**) for 209 models generated for 19 endpoints (on the X-axis). The threshold MCC value of 0.20 is marked by the dashed horizontal line. Median value of quality metrics for each endpoint is given by horizontal line in each box.

Distributions of results of each endpoint are present in Figure 2 by boxplots. Endpoints such as embryo survival (MORT) and changes in developmental defects as they relate to the whole embryo (ActivityScore) are considered as apical (robust) endpoints which gather all exposure effects at organism level consequently increasing their relevance.

Even though most of the models in this study show a relatively low MCC, this is not uncommon in biological studies. A recent study by Idakwo et al. [34] on the Tox21 data set, which became a popular data set for many QSAR and machine learning experiments [35,36,37], shows that some of the toxicological endpoints even when conducted on cell lines can have even negative values for MCC. It is therefore not unexpected that whole organism toxicity at low concentration ranges is hard to model given the MCC metrics which is expected to be more sensitive considering other often employed metrics, such as accuracy, BA, or real accuracy.

**Figure 3:**
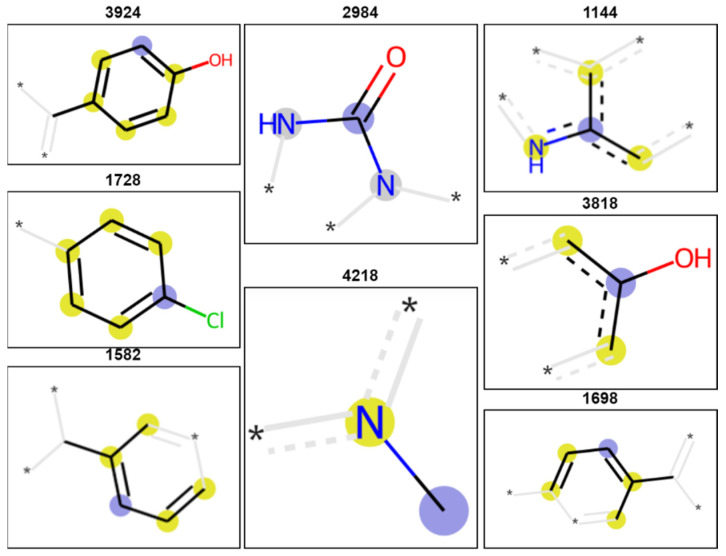
Structural fragments presented by fingerprints utilized in the final model for the JAW endpoint. The purple circle denotes the center of the fingerprint with a radius which involves atoms denoted by the yellow-colored circles. The asterisk denotes a continuation of the structure.

Out of the best models per endpoint which passed the threshold (JAW, MORT, ActivityScore) we have chosen one model each (with the highest average MCC CV and MCC Test) for interpretation. The best model for the endpoint JAW was trained on Morgan fingerprints as molecular features. It is a logistic regression model with a MCC CV of 0.20 and a MCC Test of 0.27. The average permutation importance for this model returned 10 fingerprints as the most important. The structural background (meaning) of 8 out of 10 most important fingerprints is illustrated in Figure 3. Fingerprints are bit-wise strings with zeroes and ones which are folded to a fixed length [38]. Even though they work well in building QSAR models [39,40], the folding procedure can introduce bit collision [40,41] meaning that different sub-structural fragments can be assigned to the same position in the vector. As we observe this in our own work we followed recommendations to keep a longer vector and shorter radius [41]. Nevertheless, interpretation of fingerprints should be taken with caution. In our case 2 string positions out of the 10 are heavily colliding and not presented in Figure 3. The other two chosen models for MORT and ActivityScore are both trained on RDKit descriptors. The top 10 descriptors chosen by permutation importance for the two models are listed in Table S2. For MORT (MCC CV = 0.21, MCC Test = 0.40), which was based on the Random forests classifier, amongst the most important features appear physico-chemical descriptors such as MolLogP or total polar surface area (TPSA) describing solubility and permeability of compounds through cell membrane or the number of heavy atoms in a molecule. ActivityScore (MCC CV = 0.23, MCC Test = 0.25) models is also based on the random forests classifier and among the most important features (molecular descriptors) appear the total number of NO, pyridine, aryl-COO, alkyl-OH, aryl-OH, and C(O)O groups, as well as of H-bonds acceptors (see more in Table S2). A dominance of the Random forests algorithm is seen in our previous work as well [42,43].

Other biologically relevant effects (in specific sublethal endpoints) are less often identified at low or very low concentrations being highly specific and focused on targeted changes which consequently reduce their statistical significance, as confirmed in our analyses.

Using the same methodology, we performed the modelling as the one applied on 19 CompTox endpoints presented above on four selected Tox21 endpoints related to cell-lines toxicity of 6000–7500 compounds. For each endpoint we developed model combinations based on three algorithms (RF, MLP, and LR) using structural fingerprints (FP and DS) as features. Then, two additional models for each endpoint were developed by RF algorithm on data set of physico-chemical descriptors (DS) with and without feature selection. The data set was randomly split into the training (80% compounds) and external test set (20% compounds). The obtained results are summarized in Table S3. All developed models for four Tox21 endpoints and data set of compounds have significantly higher values of MCC (training set: 0.53–0.90; test set: 0.31–0.71) and BA (training set: 0.72–0.97; test set: 0.60–0.80) both for the training and for the test set than in the cases of modeling of 19 endpoints from the CompTox data set of compounds (MCC Test between -0.085 and 0.4). It should be emphasized here that the evaluation metrics values obtained on the Tox21 set were calculated on a 6–7 times larger data set (thus being of higher significance) than the corresponding values for the CompTox set.

However, there is a need to test the quality of obtained Tox21 models in comparison with to other QSAR models performed on the same data set. In 2014, Tox21 Data Challenge [44] was organized in the prediction of 12 Tox21 endpoints. A total of four endpoints we selected for this part of modeling in order to verify the correctness of modelling methodology and the usefulness of sets of structural features (DS and FP) applied to CompTox data are from the Tox21 set of data. We compare our results with four participants [31,33,34,37] on that Tox21 challenge (Table S3), and among them are the results of the winning solutions [37], as well as the second-ranked group [33]. We were able to reproduce two metrics calculated and used in the display of results on the CompTox set, i.e., MCC and BA calculated for the test set. The data set used for training and validation of models was not standardized. Different groups designed and applied different procedures for standardization and cleaning chemical structures in the Tox21 database. Because of that, the training sets used were different in size and the compounds involved. All models developed during the competition were evaluated on the same external set of 647 chemical compounds (approximately 10 % of the data set). In order to have a more robust external (never seen) test set, we decided in our approach to take 80% of data for the training set, and the rest for the test set. (1200–1500 compounds). Thus, our external set for validation of predictive abilities of models is twice as large as in the models with which they are compared [31,33,34,37]. This means that, with a similar value of individual evaluation metrics, the reliability of the parameter related to our method is higher than the method with which we compare here. By MCC Test values our results for four Tox21 endpoints are 0.71, 0.63, 0.37, and 0.57 (Table S3), what is higher than the corresponding values obtained by Abdelaziz et al. 0.25, 0.08, 0.36, and 0.59 (respectively) [33], which are second the best overall results on the Tox21 Data Challenge. Moreover, MCC Test values obtained in this study are noticeably higher than in the study by Idakwo et al. [34] (0.29, 016, 0.62, and 0.55) and for endpoint no. 3 in Uesawa et al. [45] being 0.5 and 0.48 for two cases of dichotomization of toxicity of endpoint SR-MMP (Stress response panel - mitochondrial membrane potential). The comparison of our results with the corresponding models developed by other methodologies [31,33,34,37] by the BA gives analogous results (Table S3). Our results are 0.79, 0.73, 0.70, and 0.77 being completely comparable with the results obtained by the Tox21 Data Challenge winner [31] which are 0.74, 0.65, 0.73, and 0.9, respectively. An important characteristic of models from the study by Abdelaziz et al. [33] given in Table S3 is that, for each property, the best result is selected among 1023 models developed. In the modeling, 10 data sets of molecular descriptors are calculated and used together with other modeling options used in optimization of associative neural networks (ASNN) which were used as the algorithm in ref. [31].

Based on the results of this comparative analysis we can conclude that the methodology applied and sets of structural features calculated and used in modeling 19 CompTox endpoints are correct/valuable and correctly applied in modeling. Therefore, it seems correct to conclude that, within the methodology used and the set of structural features, the CompTox set is poorly modelable set of compounds and endpoints.

## 3. Discussion

Our results show that only three endpoints/targets (ActivityScore, MORT, JAW) can be modeled with a reasonable quality (reported with MCC > 0.20), thus promising that predictions could be above the random correlation level. In addition, the correctness of applied modeling methodology is confirmed by the comparative analysis with other studies in modeling four larger sets of Tox21 endpoints related to cell-toxicities of chemicals. An absolute value of correlation coefficient in the range 0.0 to 0.19 is characterized by many researchers as very weak, then as weak (0.2–0.39), moderate (0.4–0.59), strong (0.6–0.79), and very strong (0.8–1.0) [46,47]. Thus, because MCC is a variant of the correlation coefficient customized for classification variables, if MCC is in the range 0.0 to 0.19, such a correlation can be considered as close to random. However, if variables in correlation are medium to large like in the case of training and test sets analyzed in this study, then even the lower value of the correlation coefficient can be significant. The presented results show also that Accuracy and BA do not suffice to report classification results in imbalanced scenarios and the quality metrics such as the Cohen’s Kappa Score, MCC, and RA must be employed when reporting results on imbalanced sets of data related to toxicity [27]. The recently proposed parameter RA gives important information about the real contribution introduced by the models which are above the random accuracy level. Given as percentage, it shows the percentage contribution of the model to the total accuracy (Accuracy). One can see that all models having MCC > 0.2 have RA > 0. The models developed by [11,12] show comparingly higher MCC values on the ZF developmental endpoint provided by Padilla et al. [4], i.e., the TERATOSCORE at 144 hpf. The results are difficult to compare not only due to the endpoints being different and a lower number of compounds, but also possibly due to the different paradigms applied in data splitting. The mentioned papers utilized techniques such as diversity picking and Kennard–Stone. In our previous work, we suggest that the use informative splitting instead of random splitting can lead to optimistic generalization on external sets [43]. 

Only few QSAR methods are available to evaluate developmental toxicity [11,12,48,49,50] and the general lack of quantitative models further justifies our scope in assessing the quality and applicability of CompTox ZF model to predict outcomes on developmental endpoints. The main advantage of models such as those presented is the rapid and simultaneous toxicity prediction of numerous chemicals based on their action on development even though its applicability domain is restricted to only organic compounds. Furthermore, zebrafish dechorionated embryos (CompTox ZF; [18]) are more sensitive to chemicals exposure in comparison to chorionated embryos. The presence [51,52,53] or absence [18,21,54,55] of the chorion is important because it acts as a moderator of chemicals’ contact to embryos and their biological response. Chorion removal increases embryos sensitivity, which is an important trait for chemicals hazard identification using this assay [21]. However, previous reports confirm that the CompTox Zebrafish embryo assay might be difficult to model due to the embryo over-sensitivity that induces high control mortality [20] which is also confirmed by our results highlighting higher model quality only for apical endpoints that sum all negative events such as: Mortality and ActivityScore. Even though dechorination is desirable and promoted, it should be considered that the process of chorion enzymatic removal with pronase probably poses additional stress for ZF embryos [56]. The effect of chorion on developmental toxicity in ZF embryos has previously been investigated following chemical exposure reporting only the effects on phenotypic mortality and morphological traits [21,57]. Recently, researchers found [22] that chorion removal increases embryonic toxicity at the phenotypic level in zebrafish embryos exposed to chemicals adding potential negative effects of dechorination. Taken together, our results suggest that, similar to conclusions by [18] and [58], CompTox ZF assay using embryos survival and overall developmental gross malformations as apical endpoints could help the identification and prioritization of chemicals for more specific, targeted, and MoA-driven testing using ZF embryos as designated model organism.

## 4. Materials and Methods

### 4.1. Data Set and Chemical Representation

The data was obtained for 1092 compounds (by ID) from the US EPA CompTox Chemicals Dashboard [1]. The zebrafish embryos were assessed for 18 endpoints including yolk sac edema (YSE) and pericardial edema (PE); body axis (AXIS), trunk length (TRUN), caudal fin (CFIN), pectoral fin (PFIN), pigmentation (PIG), and somite (SOMI) deformities; eye (EYE), snout (SNOU), jaw (JAW), and otolith (OTIC) malformations; gross brain development (BRAIN); notochord (NC) and circulatory (CIRC) deformities; swim bladder presence and inflation (SWIM); touch-responses (TR) and ActivityScore, which represents a cumulative score in the database 18 above mentioned endpoints. The description of the final data set is provided in Table 2. 

The data set is heavily imbalanced with 13 to 187 active compounds per endpoint, in contrast to 812 to 977 inactive compounds per endpoint.

The data was indexed by the DTXSID and was crosschecked with the SMILES structure mappings. At first, we removed structures which did not have valid SMILES or IDs (15 compounds). Validity of SMILES was checked by the possibility to convert structures to the MOL format [59]. Furthermore, we removed duplicates by ID (19 c.) or SMILES (26 c.). We removed inorganic compounds (7 c.) and metal-containing compounds (7 c.). In the final data set compound were standardized by means of the ChemAxon Standardizer (Marvin/JChemv20.9.0, ChemAxon, Budapest, Hungary). The procedure is inspired by [60] to keep the active part of the compound. The processed data set consisting of 1018 compounds is given [61]. Molecular descriptors (2D, 3D) (DESC) and Morgan fingerprints (FP) for the predictive tasks were calculated for the 1018 structures by means of the RDKit library [62]. The fingerprint vector length was set to 5120 bits and radius to 2, i.e., the distance of 2 bonds in atom neighborhood are considered.

### 4.2. Machine Learning Methods

We employed three different classifiers in our work: Logistic Regression (LR) [63], Multi-Layer Perceptron (MLP) [64] and Random forests classifiers (RF) [65]. Logistic regression is a classification algorithm (prediction of a binary variable) which is mainly applied in linearly separable problem, even in a multidimension setting. The regression coefficients defining the boundaries of the target classes in feature space are learned from the data and penalized in this work (L^1^-norm penalty). The hyperparameters to be optimized are usually the regression coefficients (weights, bias) and the penalty. RF is an ensemble classifier. Ensemble classification algorithms are following a paradigm where multiple “weak classifiers” are trained and aggregated to improve the prediction capabilities and lower the prediction error. The weak learners here are decision trees and the aggregation is conducted by means of bootstrapping (each tree trained on a part of data and subset of features) and final voting. RF is considered a non-linear method. The hyperparameter for RF can be large and complex. Commonly optimized hyperparameters are tree depth, number of trees, class-weights, and the number of features utilized. The MLP is a fully-connected neural network. Neural networks machine learning algorithm where multiple learners are connected in layers. The learners (neurons) learn parameters (weights, bias) from the data and are “activated” by means of a non-linear function such as the sigmoid function. Hyperparameters which are commonly optimized in MLP are the number of layers, penalty function, learning rate and activation function.

The models were trained using the library scikit-learn [66] based on our previous work [42]. Since the endpoints data are imbalanced, we employed penalization and optimization techniques of the model hyperparameters to improve classification outcomes. The data were randomly split into the training (80%) and test set (20%). 

To unbias models for misclassification of the minor class (i.e., toxic compounds) during model training we employed the Matthews correlation coefficient (MCC) [28,34] as a scoring function during the model optimization. MCC is defined by Equation (1), where TP, TN, FN, FP are the elements of confusion matrix given in Table 3.

(1) $$\mathrm{MCC}=\;\frac{\mathrm{TP}*\mathrm{TN}-\mathrm{FP}*\mathrm{FN}}{\sqrt{\left(\mathrm{TP}+\mathrm{FP}\right)*\left(\mathrm{TP}+\mathrm{FN}\right)*\left(\mathrm{TN}+\mathrm{FP}\right)*\left(\mathrm{TN}+\mathrm{FN}\right)}}$$

Furthermore, we have also utilized ΔQ_2_ [30] expressed, which is named here Real-Accuracy (RA) and defined by Equations (2) and (3):

(2) RA=Accuracy−Random accuracy

(3) $$\mathrm{RA}=\frac{\mathrm{TP}+\mathrm{TN}}{\mathrm{TP}+\mathrm{TN}+\mathrm{FP}+\mathrm{FN}}-\;\frac{\left(\mathrm{TP}+\mathrm{FN}\right)*\left(\mathrm{TP}+\mathrm{FP}\right)+\left(\mathrm{TN}+\mathrm{FN}\right)*\left(\mathrm{TN}+\mathrm{FP}\right)}{{\left(\mathrm{TP}+\mathrm{TN}+\mathrm{FP}+\mathrm{FN}\right)}^{2}}$$

Balanced accuracy (BA) is given by Equation (4).

(4) $$\mathrm{BA}=\;\frac{\mathrm{TP}*\;\left(\mathrm{TN}+\mathrm{FP}\right)+\;\mathrm{TN}*\left(\mathrm{TP}+\mathrm{FN}\right)}{2*\left(\mathrm{TP}+\mathrm{FN}\right)\left(\mathrm{TN}+\mathrm{TP}\right)}$$

BA is a highly popular classification model quality measure used in QSAR studies [67]. In addition, the Cohen Kappa score [29] and other classification metrics for results evaluation [68] are also reported in Supplementary Materials (Table S1, since highly imbalanced sets (models) tend to be randomly classified [30]). 

### 4.3. Modelling

The training set was used for Bayesian hyperparameter optimization (BO) [43,69] by means of ten-fold cross-validation (10 × CV). The hyperparameter spaces are given in Appendix A for the three algorithms. BO was utilized with MCC as a loss function. BO aims to construct a posterior distribution of functions (Gaussian process) that best describes the loss function. With a growing number of observations, the posterior distribution becomes narrower, and the algorithm becomes more certain of which regions in the hyperparameter space are worth exploring further. In the process of parameter optimization, the model is continuously re-trained within the train, and the MCC results obtained by each parameter combination are evaluated. Finally, the optimal hyperparameter combination is obtained when a stopping criterion is reached (predefined number of iterations which is 20 in this work). 

For dealing with data imbalance we employed two strategies: (1) we have changed the default scoring method during cross-validation which is accuracy [66] to MCC. This means that the model penalizes cross-validation with a more sensitive metric towards imbalance. In our preliminary studies this showed a significant performance improvement comparing to default metrics; (2) we used weighting (class weights) in logistic regression and random forest. The weight ratios which are parts of the hyperparameter tuning (see Appendix A) ranged during the cross-validate hyperparameter optimization from 1:1–1:30 (rare class being 30). In our prior experiment under- and over-sampling, commonly applied in imbalanced settings, also in our previous work [42,70] did not show any results in the models trained here. The final model, described in Section 4.2, have weight ratios range from 1:8.0 (JAW) to 1:14.4 (ActivityScore).

Feature selection was performed by means of stepwise post-hoc permutation importance which showed beneficial properties independent on the basic modeling algorithm applied [43]. The permutation importance was conducted 10x per model to return an average weight. We refer to the set of options applied and considered in the model optimization, i.e., modeling algorithm used (classifier), feature selection, chemical representation and scaling as to “model combinations” which are shown in Table 4. 

This gives 11 distinct model combinations for each of the 19 endpoints, which finally yields 209 separate machine learning models. 

## 5. Conclusions

Our research provides insight into the CompTox Zebrafish embryo assays, one of the largest publicly available and most diverse data set on zebrafish aquatic toxicity. We showed that even though there are unique 1018 compounds available, the endpoints are not easy to model by the given chemical features which were utilized here (RDKit physico-chemical descriptors and Morgan fingerprints) by using three commonly used classification algorithms (Multilayer perceptron, Random forests, and Logistic regression). Molecular features used here are often used in QSAR modeling and they were calculated by the open software (RDKit). Moreover, we used three open-software classification algorithms for development of QSAR models and, consequently, this methodology can be reproduced by other authors on these data, but also applied in modeling on other problems and sets of chemicals especially in ERA of polluted aquatic ecosystems. In comparison with other methods on four Tox21 data sets and endpoints comparable (and in some cases even better) models are obtained by the methodology described in this study and applied in modeling of CompTox endpoints, thus confirming its validity. Only three out of 19 endpoints show presence of models above of “slight agreement/correlation” space defined by means of a Matthew’s correlation coefficient values (training CV and test set) above 0.20. These three models are ActivityScore, mortality (MORT), and jaw deformation (JAW). We suggest that amongst the limitations might be the experimental methods since ActivityScore and MORT are endpoints that can be predicted somewhat better by developed QSAR models as well as stress-inducing chorion removal. Other endpoints are more difficult to observe even with high-throughput screening and, consequently, it is harder to obtain a good prediction for them by developed QSAR models on the full data sets. 

## Figures and Tables

**Table 1:** Pearson correlation coefficients between quality metrics obtained for the test set across the 209 models.

	Real Accuracy	MCC	Cohen’s Kappa	Accuracy	Balanced Accuracy
Real Accuracy	1	0.84	0.86	−0.39	−0.28
MCC	0.84	1	0.97	−0.24	−0.19
Cohen’s Kappa	0.86	0.97	1	−0.18	−0.21
Accuracy	−0.39	−0.24	−0.18	1	0.59
Balanced Accuracy	−0.28	−0.19	−0.21	0.59	1

**Table 2:** Data set overview sorted by the number of active compounds per endpoint. All endpoints are binary variables having only values 1 or 0 (active or inactive). The number of missing data in each endpoint is given in the last column (“missing”).

Endpoint	Negative (0)	Positive (1)	Missing Values
AXIS	882	108	28
ActivityScore	812	187	19
BRAI	930	60	28
CFIN	942	48	28
CIRC	972	18	28
EYE	913	77	28
JAW	881	109	28
MORT	884	115	19
NC	977	13	28
OTIC	949	41	28
PE	874	116	28
PFIN	936	54	28
PIG	945	45	28
SNOU	883	107	28
SOMI	952	38	28
SWIM	958	32	28
TRUN	934	56	28
TR	912	78	28
YSE	867	123	28

**Table 3:** Elements of confusion matrix.

	Positive (Model) (1)	Negative (Model) (0)
Positive (Experimental) (1)	TP	FN
Negative (Experimental) (0)	FP	TN

**Table 4:** Model combinations presented in this work.

Classifier	Feature Set	* Scaling	** Feat. Sel.	Endpoints
Logistic regression	Fingerprints	No	No	19
Multilayer perceptron	Fingerprints	No	No	19
Random forest	Descriptors	No	No	19
Random forest	Descriptors	No	Yes	19
Random forest	Fingerprints	No	No	19
Logistic regression	Descriptors	Yes	No	19
Logistic regression	Descriptors	Yes	Yes	19
Multilayer perceptron	Descriptors	Yes	No	19
Multilayer perceptron	Descriptors	Yes	Yes	19
Random forest	Descriptors	Yes	No	19
Random forest	Descriptors	Yes	Yes	19

* Scaling = standardization of features by removing the mean and scaling to unit variance, ** Feat. Sel. = Feature Selection.

## Data Availability

The processed data used in this study is available online at [61].

## Supplementary Materials

A table with full classification results is available in Table S1, more details about the molecular features involved in the best selected models are given in Table S2. Information about QSAR models developed on Tox21 data set and details of comparison with other models developed on selected Tox21 endpoints are given in Table S3.

## Appendix A

The hyperparameters for the optimization procedure using Scikit-learn [66] are given here:

Random forests: (‘max_depth’: (5, 10), ‘n_estimators’: (70, 300), ‘max_samples’: (0.35, 0.5), n_samples_split’: (5, 10), ‘class_weight_ratio’: (5, 30));

Logistic regression: (‘l1_ratio’: (−4, 0), ‘C’: (−2, 0), ‘class_weight_ratio’: (1, 30));

Multilayer perceptron: (‘hidden layer 1’: (100, 500), ‘hidden layer 2’: (20, 100), ‘hidden layer 3’: (5, 10), ‘alpha’: (−5, −2)).
